# A comparative evaluation of ChatGPT 3.5 and ChatGPT 4 in responses to selected genetics questions

**DOI:** 10.1093/jamia/ocae128

**Published:** 2024-06-14

**Authors:** Scott P McGrath, Beth A Kozel, Sara Gracefo, Nykole Sutherland, Christopher J Danford, Nephi Walton

**Affiliations:** CITRIS Health, University of California Berkeley, Berkeley, CA 94720-1764, United States; Laboratory of Vascular and Matrix Genetics, National Heart, Lung, and Blood Institute (NHLBI), Bethesda, MD 20892, United States; Intermountain Precision Genomics, Intermountain Healthcare, St George, UT 84790-8723, United States; Intermountain Precision Genomics, Intermountain Healthcare, St George, UT 84790-8723, United States; Transplant Services, Intermountain Medical CenterMurray, UT 84107, United States; National Human Genome Research Institute, National Institute of Health, Bethesda, MD 20892-2152, United States

## Abstract

**Objectives:**

To evaluate the efficacy of ChatGPT 4 (GPT-4) in delivering genetic information about *BRCA1*, *HFE*, and *MLH1*, building on previous findings with ChatGPT 3.5 (GPT-3.5). To focus on assessing the utility, limitations, and ethical implications of using ChatGPT in medical settings.

**Materials and Methods:**

A structured survey was developed to assess GPT-4’s clinical value. An expert panel of genetic counselors and clinical geneticists evaluated GPT-4’s responses to these questions. We also performed comparative analysis with GPT-3.5, utilizing descriptive statistics and using Prism 9 for data analysis.

**Results:**

The findings indicate improved accuracy in GPT-4 over GPT-3.5 (*P* < .0001). However, notable errors in accuracy remained. The relevance of responses varied in GPT-4, but was generally favorable, with a mean in the “somewhat agree” range. There was no difference in performance by disease category. The 7-question subset of the Bot Usability Scale (BUS-15) showed no statistically significant difference between the groups but trended lower in the GPT-4 version.

**Discussion and Conclusion:**

The study underscores GPT-4’s potential role in genetic education, showing notable progress yet facing challenges like outdated information and the necessity of ongoing refinement. Our results, while showing promise, emphasizes the importance of balancing technological innovation with ethical responsibility in healthcare information delivery.

## Introduction

Recent advancements in artificial intelligence (AI), exemplified by large language models (LLMs) like ChatGPT, have ushered in transformative possibilities across various fields, including healthcare and clinical medicine. Developed by OpenAI, ChatGPT represents a significant leap in natural language processing capabilities, generating human-like text responses across a diverse array of queries.^1^^,^^2^ OpenAI used GPT-3.5 (Generative Pre-Trained Transformer 3.5)^3^ for the public release of ChatGPT on November 30, 2022. GPT-3 was initially released in June of 2020 and was comprised of 175 billion parameters. GPT-4, the next iteration of the training model is said to have 170 trillion parameters, a near 100-fold increase.^4^ There are now several LLM models in the marketplace including versions like Google’s Gemini (formerly called Bard),^5^ Facebook’s LLaMA,^6^ Anthropic’s Claude,^7^ and others. Companies developing these models face a strategic choice between open-source release, which promotes transparency and collaborative innovation, and proprietary, closed access, which secures intellectual property but limits knowledge-sharing and scientific scrutiny. This decision not only affects the accessibility of resources for researchers but also impacts the broader innovation ecosystem and ethical considerations within the field.^8^ The two leading search engines have already deployed public interfaces with AI/LLM for their platforms.^9^ Thus, consumers who may make a medical query via these portals (Microsoft’s Copilot and Google’s Gemini) are starting to receive results utilizing AI-generated responses. This also does not account for the AI offerings launched by several prominent social media platforms (Meta’s AI,^10^ Twitter/X’s Grok,^11^ and Snapchat’s My AI^12^). Incorporating LLMs into healthcare presents significant challenges, including the need for regulatory frameworks that address these models’ unique training methods and operational contexts, ensuring their safe and ethical use without compromising patient data or privacy.^13^ The FDA has requested more power to help establish guardrails and evaluate AI but would require a much larger workforce to accomplish this request.^14^ In the realm of clinical genetics and genomics, these models are especially promising, offering potential enhancements in the communication of complex genetic information.^15–17^ The versatility of ChatGPT in engaging in human-like conversations and its potential to improve patient-healthcare professional interactions are particularly noteworthy.^18^

LLMs have sparked both excitement and concern in the medical community due to their potential transformative impact and inherent risks. There have been an explosion of papers testing various facets and use cases. These models, capable of assisting in clinical documentation, summarizing research papers, and functioning as patient-interaction chatbots, have been recognized for their diverse applications in healthcare.^13^ ChatGPT, in particular, garnered attention for passing United States Medical Licensing Examinations, with its performance, and that of GPT-4, significantly surpassing its predecessors.^19^ However, despite these achievements, the use of LLMs in clinical decision-making or patient communication remains a subject of caution. This is due to instances where LLMs have failed to provide accurate information in response to patient queries or to suit individual patient circumstances, leading to the assertion that patients often cannot distinguish between information provided by LLMs and human clinicians.^20^ Both physicians and patients share concerns about generative AI. In one patient survey (*n* = 1000), 80% of respondents indicated they had a concern about generative AI in healthcare, and 89% stated a desire for clinicians to be clear and transparent about its use in medicine.^21^ In a separate survey of physicians (*n* = 1043), nearly 2 out of 3 expressed concern about AI algorithms driving diagnosis and treatment decisions and 80% stated that it is “very important” that doctors be informed on AI.^22^ LLMs are also positioned to assist with certain challenges associated with clinical genetics and genetic counseling. There has been a persistent shortage of licensed experts in the genetic counseling and clinical genetics space for some time now.^23–25^ In 2021, there were 5659 Certified Genetic Counselors (CGC), which equates to 1 clinical CGC per 100 000 population,^26^ and only 1240 clinical geneticists as of April 2020.^27^ In 2018, there were approximately 75 000 genetic tests on the market, with an estimated 10 new tests appearing per day.^28^ In 2022, that number had risen to over 129 624 in the US, and 197 779 tests available globally.^29^ Studies have shown that public knowledge and literacy gaps about clinical genetics have long existed.^30^^,^^31^ Chatbots have been pursued as a mechanism to address the staffing shortages and patient knowledge gaps,^32^^,^^33^ but they often relied upon decision trees, and structured AI models, where patients were guided towards pre-written responses to their queries. Generative AI models introduce a novel mechanism to assist patients with their genetic and genomic questions.

ChatGPT has shown potential in enhancing healthcare delivery, patient education, and influencing patient compliance through improved accessibility and communication.^18^^,^^34^ However, these applications come with inherent limitations and ethical concerns, including the accuracy and reliability of AI-generated medical advice and the risk of misinformation.^15^ The ability of ChatGPT to reproduce human language convincingly raises important questions about its use in clinical diagnostics and decision-making, underscoring the need for extensive training and formal evaluation against standard clinical practices.^18^^,^^35^ Some of the significant challenges that face LLM integration in genetic education include the following:

- Data privacy and security^36^
- Accuracy and reliability^37^
- Integration with existing systems^38^
- Ethical and legal considerations^39^
- User trust and acceptance^40^
- Training and education^41^
- Bias and generalizability^20^
- Regulatory approval and oversight.^13^

In our earlier study^17^ of ChatGPT 3.5’s (December 2022 model) ability to work with genetic queries, the model showed promise and areas for improvement. It correctly answered 64.7% of genetic queries, showed proficiency in context recognition (Bot Usability Scale [BUS-15]^42^ score: 5.5/7), and provided moderately relevant and informative responses (average BUS-15 scores: 5.5/7 and 5.0/7, respectively). However, it was less adept at handling varied inputs (score: 3.0/7) and needed improvement in clarifying complex genetic concepts (score: 5.75/7).

This study seeks to further explore the capabilities of ChatGPT, focusing on its latest iteration, ChatGPT 4 (March 23, 2023 model). Building on our previous work with ChatGPT 3.5 (GPT-3.5), which demonstrated promising capacity for providing responses to queries about genetic disorders, but showed some significant limitations,^17^ this study delves deeper into the nuanced applications and challenges of ChatGPT 4 (GPT-4) in a clinical setting. We seek to assess not only the utility but also the limitations and ethical implications of using ChatGPT in medical settings. The outcome of this study is intended to contribute to the growing discourse on the practical applications of AI in healthcare and to guide future developments in the field.

## Methods

Refer to the Supplementary method for additional details in the following areas.

### Study design

This study was conducted to evaluate the performance of GPT-4 (March 23, 2023 Model) in the context of genetic counseling and education. The evaluation involved a structured survey, which included questions selected from the Bot Usability Scale (BUS-15)^42^ and additional custom questions designed to assess the clinical value of GPT-4’s responses to questions about 3 genes patients may ask providers: *BRCA1* (OMIM 113705), *HFE* (OMIM 613609), and *MLH1* (OMIM 120436).

### Survey development

Informed by our observations from the GPT-3.5 study, we refined our survey to enhance its focus and relevance. Initially composed of 12 questions, the survey was reduced to 9, implemented via Qualtrics. This revision included 7 questions directly selected from the BUS-15, complemented by 2 additional questions that we specifically designed to assess the quality and relevance of the chatbot’s responses. The first 2 questions were as follows:

1. The overall quality of the Chatbot’s response is (*5-point Likert: Very poor to Very Good*)
2. The Chatbot delivered an answer that provided the relevant information you would include if asked the question. (*5-point Likert: Strongly disagree to Strongly agree*)

From the BUS-15’s full set of questions, we selected 7 items that most directly align with our research objectives; questions not relevant to the present study were omitted (eg, ease of use and response time).

The BUS-15 questions (*7-point Likert: Strongly disagree to Strongly agree*) focused on:

1. *Recognition and facilitation of users’ goal and intent*: Chatbot seems able to recognize the user’s intent and guide the user to its goals.
2. *Relevance of information*: The chatbot provides relevant and appropriate information/answer to people at each stage to make them closer to their goal.
3. *Maxim of quantity*: The chatbot responds in an informative way without adding too much information.
4. *Resilience to failure*: Chatbot seems able to find ways to respond appropriately even when it encounters situations or arguments it is not equipped to handle.
5. *Understandability and politeness*: The chatbot seems able to understand input and convey correct statements and answers without ambiguity and with acceptable manners.
6. *Perceived conversational credibility*: The chatbot responds in a credible and informative way without adding too much information.
7. *Meet the neurodiverse needs*: Chatbot seems able to meet needs and be used by users independently form their health conditions, well-being, age, etc.

### Gene selection and question design

In developing the initial query prompts, 3 experts (1 clinical geneticist, and 2 genetic counselors) created 68 questions, focusing on BRCA1, MLH1, and HFE. These genes were chosen due to the relative frequency with which they are observed in clinical practice and their differing inheritance patterns—autosomal dominant for BRCA1 and MLH1, and autosomal recessive for HFE. This selection provided a comprehensive overview of potential genetic counseling scenarios. The queries were designed to reflect typical patient inquiries, ranging from straightforward to complex and subjective questions.

### Expert panel and data collection

A panel of experts (2 genetic counselors and 2 clinical geneticists) were provided a link to the survey questions. The team of domain experts were selected due to their experience working in a large-scale program to return actionable genetic findings to patients^43^ and an expert outside of the group was also recruited to provide an additional perspective. This was the same panel of experts who participated in the GPT-3.5 study. They independently evaluated the responses from GPT-4 without discussing the questions or answers among themselves until after survey submission. This approach ensured unbiased evaluation.

### GPT-4 evaluation

The release of GPT-4 occurred shortly after completing the work on the GPT-3.5 study. In order to evaluate the newest model of GPT, we repeated the survey with the same set of prompts, and this time captured the new responses from the GPT-4 model. In this iteration, we focused on 2 primary aspects:

1. *Accuracy of the Answer*: Evaluating the factual correctness of GPT-4’s responses.
2. *Relevance of the Information Provided*: Assessing how relevant GPT-4’s responses were to the questions asked.

The same BUS-15 questions used in the initial study with GPT-3.5 were included to maintain consistency and allow for comparative analysis. Three questions were dropped from the qualitative analysis component from the 3.5 paper to help reduce the time for the evaluators to complete their work. The experts reported taking 5-6 hours to complete the evaluation of the 68 questions from the 3.5 study.

### Statistical analysis

Responses from GPT-4 were analyzed using descriptive statistics to understand the distribution of scores and to compare them against the benchmarks set by the expert panel using Prism. The data collected from the free-text responses provided insights into specific areas of strength and weakness in GPT-4’s performance. Statistics included a mixed-effects analysis of expert responses to evaluate ChatGPT version and question level effects. We then looked at how the different versions performed at the question level using Sidak’s multiple comparisons test. One-way ANOVA was used to evaluate for topic level differences in relevance and Wilcoxon tests were used to compare the ChatGPT versions using questions from the BUS-15.

## Results

The interactions with GPT-4 resulted in 4 chat transcripts, with 21 *BRCA1*, 20 *HFE*, and 27 *MLH1* questions, respectively, for each of the genes. *BRCA1*, *HFE*, and *MLH1* are key genes associated with significant health conditions: *BRCA1* with breast and ovarian cancers, *HFE* with hereditary hemochromatosis, and *MLH1* with Lynch syndrome, a form of hereditary colorectal cancer. The full study data can be located on datadryad.org.^44^

### Accuracy and relevance

**Figure 1.**
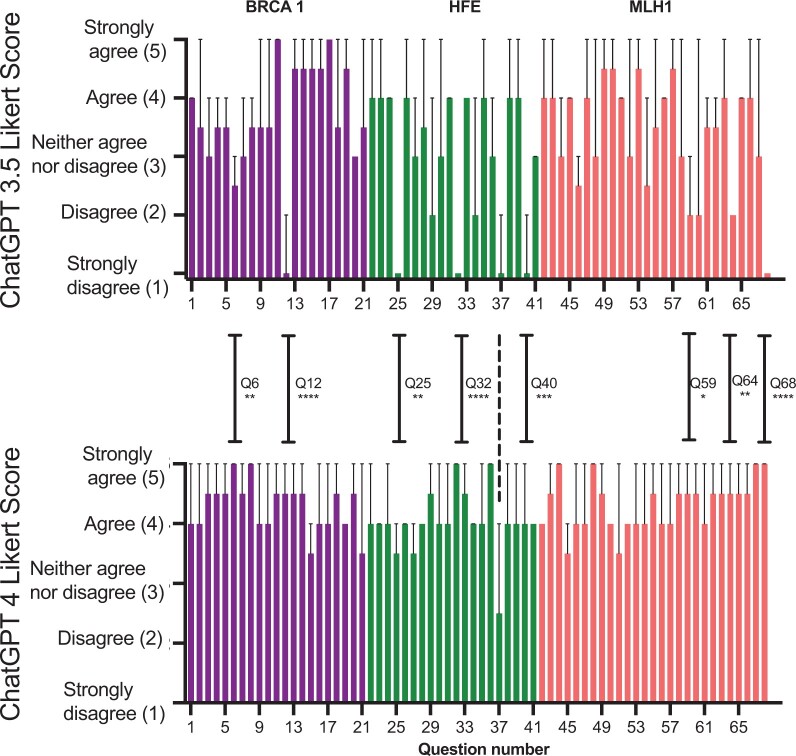
GPT-4 shows improved accuracy for genetic information as comparted to GPT-3.5. The top graph shows the median and 95% confidence interval (CI) for accuracy ratings for the GPT-3.5 responses to the 68 prompts, while the bottom graph shows median and 95% CI for ChatGPT 4. Questions 1-21 related to BRCA1-related breast cancer; questions 22-41 correspond to HFE-related hemochromatosis and responses 42-68 are regarding MLH1-related Lynch syndrome. A mixed model analysis showed both a version effect (*P*<0.0001, with GPT-4 performing better) and a question level effect (*P*<0.0001), with a corresponding interactive effect (*P*<0.0001). Statistically significant different responses (Sidak) between the 2 versions are noted by solid bars while the hashed bar represents a low scoring question in both sets.

In order to evaluate the quality of the responses on accuracy (GPT-3.5 and 4) and relevance (GPT-4 only), experts were asked to grade each answer and rank it on a 5-point scale (1 = very poor, and a 5 = very good). We compared the first question from the GPT-3.5 survey with the first question from the GPT-4 survey for accuracy and analyzed the second question in the GPT-4 survey for relevancy. The overall accuracy from the 3.5 model was 3.38 (95% CI: [3.23, 3.53]) and the GPT-4 model was 4.17 (95% CI [4.08, 4.26]), Figure 1. A mixed effects analysis showed both a ChatGPT version effect (*P* < .0001) and a question level effect (*P* < .0001) as well as a version by question interactive effect (*P* < .0001). General improvement in accuracy scores was noted across all 3 gene categories (Figure 1).

Multiple comparisons testing evaluating each question in both versions using Sidek’s multiple comparisons framework, showed statistically significant improvement in scores for 8 questions.

- Q6 Does having a *BRCA1* mutation affect the treatment of cancer?
- Q12 I have a *BRCA1* mutation, is there any chance I could have given this to my spouse?
- Q25 What are the chances that my son will have hemochromatosis?
- Q32 Are men more likely than women to have the hemochromatosis mutation?
- Q40 How do I know which parent I got the hemochromatosis mutation from?
- Q59 Is there anything I can do to make sure my children do not inherit my *MLH1* gene mutation?
- Q64 If my uncle has an *MLH1* mutation what is my risk of having it?
- Q68 My family has Lynch syndrome, but I don’t have it. Can I still get cancer?

Overall, these “most improved” questions made up 8 of the lowest scoring 9 questions in the GPT-3.5 model. Seven of them pertain to queries regarding inheritance patterns. One originally low-scoring question (Q37), “How do I make sure I don’t pass the hemochromatosis mutation to my children?” continued to score poorly in both models.

**Figure 2.**
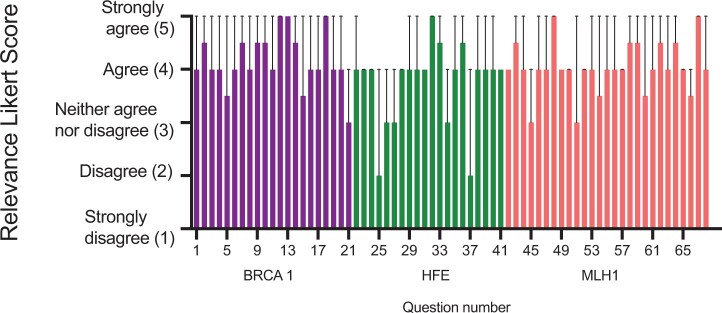
Relevance scores for genetic education questions in ChatGPT 4. The presented graph shows the median and 95% CI for relevance ratings for the GPT-4 responses to the 68 prompts. Questions 1-21 refer to BRCA1-related breast cancer; questions 22-41 correspond to HFE-related hemochromatosis and responses 42-68 are regarding MLH1-related Lynch syndrome. Although the ratings vary by question, there is no statistically significant difference by condition (1-way ANOVA).

For the GPT-4 model, we also asked each expert to evaluate if the chatbot provided relevant information based off the prompts and responses. A 5-point Likert scale was used (1= Strongly disagree, 5 = Strongly agree). The mean score of GPT-4 for relevance of responses was 3.92 (95% CI [3.86, 3.98], median = 4.0. When broken down by gene *BRCA1* had a mean of 4.11 (95% CI [4.06, 4.16], median = 4.0), *HFE* mean was 3.70 (95% CI [3.55, 3.85], median = 4.0), and *MLH1* mean was 3.94 (95% CI [3.89, 3.99], median = 4.0) (Figure 2). These scores were not statistically different by 1-way ANOVA.

### Understandability and conversational tone

**Figure 3.**
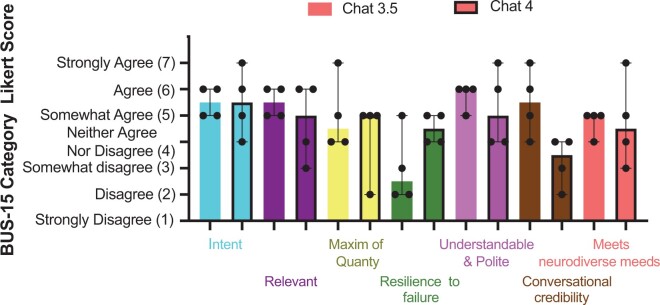
Understandability and conversational tone trends downward in ChatGPT 4. Median with 95% CI for the question level responses from the selected BUS-15 questions for both GPT-3.5 and GTP-4. Values for GPT-4 in each category are shown with black outline and those for GPT-3.5 are shown with no outline.

Scores for the BUS-15 subset are shown in Figure 3 (7-point Likert scale: 1 = strongly disagree to 7 = strongly agree). Overall scores on the BUS subset, either by total score (GPT-3.5 median = 35.5 IQR = 5.5; GPT-4 median = 32.0 IQR = 4.75) or by individual category (Figure 3) did not differ between ChatGPT versions. A downward trend, however, was noted in both. Both versions scored well (agree or better) in their ability to recognize the user’s intent and facilitate their goals (Figure 3, turquoise) and understandability and politeness (Figure 3, purple), but most other areas were closer to neutral/somewhat agree, especially in the GPT-4 model. Chat 3.5 appeared to score lower on conversational credibility (Figure 3, brown); however, the difference was not statistically significant by the Wilcoxon Matched Pairs Rank test. Likewise, the apparent resistance to failure that appeared to improve in GPT-4 (Figure 3, green) was also non-significant.

## Discussion

The evolving landscape of AI, particularly with models like ChatGPT, has continued to show great potential across various domains,^20^^,^^45–47^ including genetics.^48^^,^^49^ While excitement surrounds these advancements, concerns about potential misuse and the dissemination of incorrect information persist.^34^^,^^50^ Our study’s primary aim was to evaluate the efficacy of ChatGPT, specifically version 4.0, in accurately and effectively delivering genetic information. The comparative analysis with its predecessor, GPT-3.5, revealed significant strides in the AI’s ability to handle complex genetic topics, albeit with areas necessitating caution.

### Improvements in GPT-4 over 3.5

Our comprehensive analysis revealed nuanced improvements in GPT-4 compared to its predecessor. In our analysis, GPT-4 demonstrated marked improvements over version 3.5 in aspects of accuracy (Figure 1, *P* < .0001). While overall enhancements were notable, the analysis revealed significant variability in performance. An interactive effect (*P* < .0001) was observed, suggesting that the accuracy of the responses is influenced by both the ChatGPT version, and the specific question being asked. In addition, the BUS-15 scores generally declined from GPT-3.5 to GPT-4 suggesting a trend toward decreased understandability of the later version (Figure 3). While not statistically significant, in discussion after the survey was completed, the reviewers generally found the scripting in GPT-4 to be more correct but less natural and more prescriptive overall.

These advancements suggest an evolution of GPT-4’s capabilities but underline the necessity for ongoing development to meet the specialized demands of genomic information delivery. For instance, we wish to call to attention question 37. This question did particularly poorly in 3.5, and while it improved in 4, it still was a problematic answer. The recognition of inheritance patterns was improved overall in 4, but there was no consideration of penetrance when discussing whether the offspring would be affected by hemochromatosis. Question 37 (Table 1), marked by its lack of progress, illustrates how even upgraded models can retain blind spots or inaccuracies. This underscores the necessity for continuous refinement in AI models and the importance of human oversight, especially in complex domains like healthcare. GPT-4’s coherent engagement in conversations about genetic conditions is promising, yet continuous updates and expert oversight are critical to its application in healthcare. The current business model of OpenAI offers the 3.5 model for free, where there is a monthly charge to access the 4.0 model. That barrier might be enough for patients to opt for 3.5 rather than paying for the enhanced 4.0 model. As we found in our prior study, GPT-3.5 was startling in its ability to communicate conversationally about health, but requires further refinement, as documented with our findings with GPT-4.

**Table 1:** Notable Responses

Question number	ChatGPT 4’s response
3) What is my risk of cancer with a BRCA1 mutation?	The exact risk of developing cancer for individuals with a BRCA1 mutation can vary depending on several factors, including family history, personal medical history, and the specific type of mutation. However, I can provide some general risk estimates for those with a pathogenic BRCA1 mutation: 1) Breast cancer: Women with a BRCA1 mutation have a lifetime risk of developing breast cancer that ranges from 50% to 80%. This is significantly higher than the average risk for women in the general population, which is about 12%. Men with a BRCA1 mutation also have an increased risk of breast cancer, but the overall risk is still low, at around 1%-2%. 2) Ovarian cancer: Women with a BRCA1 mutation have a lifetime risk of developing ovarian cancer between 20% and 40%. This is much higher than the average risk for women in the general population, which is about 1.3%. 3) Prostate cancer: Men with a BRCA1 mutation may have an increased risk of developing prostate cancer, but the exact risk is not well-established. Some studies suggest that the risk may be up to 2 times higher than in men without the mutation. 4) Pancreatic cancer and other cancers: People with a BRCA1 mutation may have a slightly increased risk of developing pancreatic cancer and other cancer types, but the overall risk remains low. It is essential to remember that having a BRCA1 mutation does not guarantee the development of cancer; it only increases the risk. Personalized risk assessments and consultations with healthcare professionals, such as genetic counselors, can help individuals with a BRCA1 mutation understand their specific risk and develop appropriate strategies for cancer screening, prevention, and management.
9) Should I tell my children if they have the same BRCA1 mutation that I do?	Deciding whether and when to tell your children about their potential BRCA1 mutation is a complex and personal decision that depends on several factors, including your children's age, maturity, and emotional readiness. In general, children may not be able to fully understand the implications of a BRCA1 mutation or be prepared to handle the associated emotional and medical aspects. Some experts suggest waiting until they reach late adolescence or early adulthood before discussing genetic testing and the potential for inheriting a BRCA1 mutation. At this stage, they may be more capable of understanding the information and making informed decisions about their healthcare. It is crucial to keep the following points in mind when considering discussing a BRCA1 mutation with your children: 1) Emotional readiness: Consider whether your child is emotionally prepared to handle the information and the potential anxiety or stress it may cause. 2) Age-appropriate information: When discussing the topic, present the information in an age-appropriate manner that your child can understand. 3) Genetic counseling: Involve a genetic counselor in the process to help guide the conversation and provide accurate, up-to-date information about BRCA1 mutations, associated cancer risks, and potential risk management strategies. 4) Ongoing support: Ensure that your child has access to emotional support, either from you or from professional resources, as they process the information and make decisions about their healthcare. 5) Respect their autonomy: Ultimately, the decision to undergo genetic testing and pursue risk management strategies should be your child's choice when they reach an appropriate age. Discussing a potential BRCA1 mutation with your children can be challenging, and it may be helpful to consult with a genetic counselor or healthcare professional for guidance on navigating this conversation and determining the best approach for your family.
10) At what age should I tell my children about their BRCA1 mutation?	Deciding at what age to tell your children about their potential BRCA1 mutation depends on several factors, such as their emotional maturity, readiness to handle the information, and the relevance of the information to their current stage of life. There is no one-size-fits-all answer, but many experts recommend waiting until late adolescence or early adulthood to discuss genetic testing and the possibility of inheriting a BRCA1 mutation. At this stage, your children are more likely to understand the implications of a BRCA1 mutation and be able to make informed decisions about their healthcare. This is also an age where cancer screening and risk reduction strategies become more relevant, as the risk of developing cancer associated with a BRCA1 mutation typically increases with age. When discussing a potential BRCA1 mutation with your children, consider the following: 1) Provide age-appropriate information that they can understand. 2) Offer emotional support and reassurance. 3) Involve a genetic counselor to help guide the conversation and answer any questions your children may have. 4) Encourage open communication and respect their autonomy in making decisions about genetic testing and risk management strategies. Ultimately, the decision of when to tell your children about their potential BRCA1 mutation should be based on your knowledge of your children and your family's unique circumstances. Consulting with a genetic counselor or healthcare professional can provide guidance on the best approach for your family.
12) Is my husband at risk of cancer if he has a BRCA1 mutation?	Yes, men with a BRCA1 mutation are at an increased risk of developing certain types of cancer, although the risks are generally lower than those for women with the same mutation. For men with a BRCA1 mutation, the most common cancer risks include: 1) Breast cancer: Although male breast cancer is relatively rare, men with a BRCA1 mutation have a higher risk of developing the disease compared to men in the general population. The estimated lifetime risk of male breast cancer for those with a BRCA1 mutation ranges from 1% to 5%. 2) Prostate cancer: Some studies have suggested that men with a BRCA1 mutation may have a higher risk of developing prostate cancer, particularly more aggressive forms of the disease. The exact increase in risk is not well-established, but it is generally considered to be modest. 3) Other cancers: Men with a BRCA1 mutation may also have a slightly increased risk of developing other cancers, such as pancreatic cancer or melanoma, but the risk is less well-defined and may vary depending on other genetic and environmental factors. If your husband has a BRCA1 mutation, it is important for him to discuss his cancer risks and appropriate risk management strategies with a healthcare professional or genetic counselor. They can provide personalized recommendations based on his specific risk factors and family history, which may include regular screenings, lifestyle modifications, or other preventive measures.
16) If I have a BRCA1 mutation can I change my genetic code with CRISPR?	CRISPR (Clustered Regularly Interspaced Short Palindromic Repeats) is a powerful gene-editing technology that has shown potential in correcting genetic mutations in various experimental models. While CRISPR has demonstrated success in laboratory settings and some clinical trials, it is important to note that its application for editing human germline cells (sperm, eggs, or embryos) to correct BRCA1 mutations remains a complex and controversial issue. There are several challenges and ethical concerns associated with using CRISPR to edit the human germline, including: 1) Safety: CRISPR technology can sometimes introduce unintended changes to the DNA, known as off-target effects, which may cause unforeseen consequences. Before this technology can be safely applied to humans, researchers need to develop methods to minimize these off-target effects and thoroughly assess the long-term safety of gene-editing. 2) Ethical considerations: The use of CRISPR for editing human germline cells raises numerous ethical questions, including concerns about the potential for designer babies, the implications of altering the human gene pool, and the possible unforeseen consequences of making permanent changes to an individual's genetic makeup. 3) Regulatory issues: Most countries currently have regulations that limit or prohibit the use of gene-editing technologies in human germline cells. It will be essential to develop a clear regulatory framework that addresses the ethical and safety concerns before the widespread application of CRISPR in this context. In summary, while CRISPR holds promise for correcting genetic mutations such as BRCA1, its application to edit human germline cells remains experimental and is currently limited by safety, ethical, and regulatory concerns. Research is ongoing, and future advances may lead to safe and ethical ways to utilize gene-editing technologies for the prevention and treatment of genetic conditions.
37) How do I make sure I don't pass the hemochromatosis mutation to my children?	The inheritance of genetic mutations, such as the one causing hemochromatosis, is determined by the combination of genes that each parent contributes to their offspring. As a result, you cannot completely guarantee that you won't pass the hemochromatosis mutation to your children. However, you can make informed decisions about family planning by understanding the inheritance patterns of hemochromatosis and your partner's genetic status. Hereditary hemochromatosis is an autosomal recessive condition, which means that an individual must inherit 2 mutated copies of the gene (1 from each parent) to be affected. If you have the hemochromatosis mutation, you are a carrier, and the risk of passing the mutation to your children depends on your partner's genetic status: If your partner is not a carrier: Each child has a 50% chance of inheriting 1 mutated gene and being a carrier, but they will not be affected by hemochromatosis. If your partner is a carrier: Each child has a 25% chance of inheriting 2 mutated genes and being affected by hemochromatosis, a 50% chance of inheriting 1 mutated gene and being a carrier, and a 25% chance of not inheriting the mutation at all. You and your partner may consider genetic testing to determine your risk of passing the mutation to your children. Consulting with a genetic counselor can help you better understand your risk, explore your family planning options, and make informed decisions. Some couples choose to pursue options such as preimplantation genetic diagnosis (PGD) in combination with in vitro fertilization (IVF) to minimize the risk of passing the hemochromatosis mutation to their children. However, these procedures can be complex, expensive, and may not be suitable for everyone. Discuss your concerns and options with your healthcare provider or a genetic counselor to determine the best course of action for your specific situation.
46) If I have Lynch syndrome will I get cancer?	Having Lynch syndrome increases your risk of developing certain types of cancers, particularly colorectal cancer and endometrial cancer, as well as other cancers like ovarian, stomach, small intestine, liver, gallbladder, urinary tract, brain, and skin cancers. However, having Lynch syndrome does not guarantee that you will develop cancer. The lifetime risk of developing cancer for individuals with Lynch syndrome varies depending on the specific gene mutation and other factors, such as family history and lifestyle. While it is important to be aware of your increased risk, you should also remember that proactive measures can help manage your risk and improve your overall health. If you have Lynch syndrome, consider the following steps to manage your risk: 1) Regular surveillance and screening: Undergo regular cancer screenings, such as colonoscopies and endometrial biopsies, as recommended by your healthcare provider. Early detection can lead to better treatment outcomes. 2) Risk-reducing surgeries: In some cases, prophylactic surgeries, such as a colectomy (removal of part or all of the colon) or hysterectomy (removal of the uterus), may be recommended to reduce the risk of developing cancer. 3) Lifestyle modifications: Adopting a healthy lifestyle, including a balanced diet, regular exercise, maintaining a healthy weight, and avoiding tobacco products, can help reduce your overall cancer risk. 4) Collaborate with healthcare professionals: Work closely with your healthcare provider, genetic counselor, and other specialists to develop a personalized management plan based on your specific risks and medical history. Taking an active role in your health and staying informed about your condition can help you better manage your cancer risk and improve your overall quality of life.
48) Is there a cure for Lynch syndrome?	There is currently no cure for Lynch syndrome, as it is a genetic condition caused by mutations in specific DNA mismatch repair genes. However, individuals with Lynch syndrome can manage their increased cancer risk through proactive measures and regular surveillance. Some of the key steps to manage Lynch syndrome include: 1) Regular surveillance and screening: Early detection is crucial for improving treatment outcomes. Follow the cancer screening recommendations provided by your healthcare provider, such as regular colonoscopies and endometrial biopsies. 2) Risk-reducing surgeries: In some cases, prophylactic surgeries, like colectomy (removal of part or all of the colon) or hysterectomy (removal of the uterus), may be recommended to reduce the risk of developing cancer. 3) Lifestyle modifications: Adopting a healthy lifestyle, including a balanced diet, regular exercise, maintaining a healthy weight, and avoiding tobacco products, can help reduce your overall cancer risk. 4) Collaborate with healthcare professionals: Work closely with your healthcare provider, genetic counselor, and other specialists to develop a personalized management plan based on your specific risks and medical history. It is important to stay informed about your condition, collaborate with healthcare professionals, and take an active role in managing your health to reduce the impact of Lynch syndrome on your life.
50) How do I tell my kids I have Lynch syndrome?	Telling your children about your Lynch syndrome diagnosis can be challenging, as it involves discussing complex genetic information and potential health risks. Here are some tips to help guide the conversation: 1) Be prepared: Before talking to your children, make sure you have a clear understanding of Lynch syndrome, its implications, and the steps you will take to manage your risk. Gather reliable resources and be prepared to answer their questions or address their concerns. 2) Choose the right time and place: Select a comfortable and quiet setting for the conversation, and ensure you have enough time to discuss the topic without interruptions. 3) Use age-appropriate language: Tailor your explanation to your children's age and understanding. For younger children, use simple and straightforward language. For older children and teenagers, you can provide more detailed information. 4) Focus on the positive: Emphasize the benefits of knowing about Lynch syndrome, such as increased vigilance, regular screenings, and the possibility of early detection and treatment for cancers associated with the condition. 5) Address their concerns: Your children may have concerns about their own risk or your health. Be open to discussing their fears and provide reassurance that you are working closely with healthcare professionals to manage the condition. 6) Discuss the possibility of genetic testing: Explain that they may consider genetic testing in the future (typically when they reach adulthood) to determine if they have inherited the Lynch syndrome mutation. Emphasize the importance of consulting a healthcare provider or genetic counselor before deciding on testing. 7) Offer support: Let your children know that you are there to support and guide them through this process. Encourage open communication and make sure they feel comfortable asking questions or discussing their feelings. Remember that this conversation is just the beginning of an ongoing dialogue. It is essential to maintain open communication and provide updates about your health and any new information about Lynch syndrome as it becomes available.
55) What is my risk of cancer with a MLH1 mutation?	If you have a pathogenic mutation in the MLH1 gene, which is associated with Lynch syndrome, your risk of developing certain types of cancer is significantly increased compared to the general population. The exact risk varies depending on the individual and family history, but some common risk estimates include: 1) Colorectal cancer: Individuals with a MLH1 mutation have a 52% to 82% lifetime risk of developing colorectal cancer, compared to around 5% in the general population. 2) Endometrial cancer: Women with a MLH1 mutation have a 25% to 60% lifetime risk of developing endometrial cancer, compared to approximately 3% in the general population. 3) Ovarian cancer: Women with a MLH1 mutation have an estimated 4% to 24% lifetime risk of developing ovarian cancer, compared to around 1.3% in the general population. 4) Gastric cancer: Individuals with a MLH1 mutation have a 6% to 13% lifetime risk of developing gastric cancer, compared to around 1% in the general population. 5) Other cancers: Lynch syndrome has also been associated with an increased risk of developing other cancers, such as small bowel, hepatobiliary, urinary tract, brain, and certain skin cancers. It is important to note that these are estimated risks and may not accurately predict an individual's chance of developing cancer. If you have a MLH1 mutation, it is crucial to work closely with healthcare professionals and genetic counselors to develop a personalized cancer risk management plan that includes regular cancer screenings, lifestyle modifications, and possibly risk-reducing surgeries.
67) I am worried about my MLH1 mutation, I’m scared I will get cancer.	It is understandable to feel worried or scared when you learn you have an MLH1 mutation associated with Lynch syndrome, as it does increase the risk of developing certain types of cancer. However, it is important to remember that having the mutation does not guarantee that you will develop cancer. There are steps you can take to manage your risk and maintain your health. 1) Consult a genetic counselor or healthcare provider: They can help you understand your specific risks, provide personalized recommendations for cancer screening, and address any concerns you may have. 2) Regular screenings: Follow a personalized cancer screening schedule, which may include more frequent or earlier screenings for cancers associated with Lynch syndrome, such as colorectal, endometrial, ovarian, stomach, and other related cancers. 3) Lifestyle modifications: Adopt a healthy lifestyle by maintaining a balanced diet, exercising regularly, avoiding tobacco, limiting alcohol consumption, and maintaining a healthy weight. These lifestyle changes can help reduce the overall risk of developing cancer. 4) Risk-reducing surgeries: In some cases, your healthcare provider may recommend risk-reducing surgeries, such as prophylactic removal of the colon or uterus, depending on your specific risk factors and personal preferences. 5) Emotional support: It is essential to address the emotional impact of having an MLH1 mutation. Reach out to friends, family, or support groups for people with Lynch syndrome to share your experiences and learn from others who are going through similar situations. Professional counseling or therapy may also be helpful in coping with the anxiety and fear associated with the increased cancer risk. Remember that knowledge about your genetic status empowers you to take proactive measures to manage your risk. Focus on the actions you can take to maintain your health and work closely with your healthcare team to develop a personalized plan for cancer prevention and early detection.

### Persistent challenges and ethical considerations

Certain challenges also persist. For instance, variability in performance, especially in providing informative responses, indicates room for further refinement. Additionally, AI’s increased reliance on scripted responses in the GPT-4 version may compromise the naturalness of interactions, a vital aspect of patient communication. Moreover, the potential of providing outdated or inaccurate information remains a significant hurdle. ChatGPT’s training on historical datasets by default means it may not include information regarding the most current clinical practices. This particular hurdle is not insurmountable as some LLM models such as Grok^11^ have demonstrated real-time knowledge acquisition capabilities. The more complicated issue here may be access to information for real-time training as the content publishers try to either prevent or monetize access to their data for training.^51^

Our reviewers stated that the new version appeared more scripted and less like a human interaction. This may explain what lead to some of the BUS-15 scores dipping from version 3.5 to 4. While we do not have access to ChatGPT’s underlying software architecture, we suspect that constraints were applied in GPT-4, leading to answers that appeared to be more scripted with a diminished conversational tone when compared to those observed in GPT-3.5 (see Questions 9, 10, 16, and 50 in full dataset^44^). We suspect that this change is likely due the placement of guardrails to increase the accuracy of the information delivered.^52^^,^^53^ This balance of delivering medically precise information in patient-friendly language is challenging to strike, especially in medical contexts where delivering accurate information is paramount; even minor inaccuracies can have significant consequences.

Upon reviewing expert flagged outputs from GPT-4, errors broadly fit 2 categories:

1. Responses that adhered to previous guidelines, which would have been accurate in the past.
2. Miscalculated or poorly referenced disease risks.

While several risk assessments offered by the chatbot raised concern (Table 1: question 3, 12, and 55), they were not classified as incorrect if they marginally aligned with accepted parameters, even if on the outer edges. Notably, one risk assessment had no identifiable basis in the literature we reviewed (Table 1: question 12). These issues underscore key challenges inherent to LLMs. Given their training on vast historical datasets, they might inadvertently prioritize older information over newer updates. Since LLMs inherently weigh more frequently appearing data more heavily, outdated information, if more prevalent in literature,^54^ might be favored. This may have been the case for the responses regarding prophylactic colectomy as a preventative treatment for Lynch syndrome, an outdated concept that appeared in 3 different answers (questions 44, 46, 48, and 59). Furthermore, topics of contention, due to their extensive debate, might unduly influence the model and inadvertently prioritize controversial perspectives. However, one can guide ChatGTP by specifying context. For instance, asking it to “share the most recent information on Twitter/X” or “offer insights post-2020” might refine its outputs, favoring newer data. Yet, this is not a natural conversational approach for most users. Also, the concept of prompt engineering, or the optimizing or refining the prompt sent to an LLM as a means to enhance the results, may be declining in effectiveness.^55^ Many generative AI models can now generate their own prompt optimization behind the scenes before submitting them for a response.^56^

Keeping LLM models up to date with the latest clinical practices is challenging given the nature of training on historical data. Methods exist to inject or train LLM’s^57^ with new information. However, administering and regulating this process raises several questions. Notably, which governing body oversees this process, and who determines the source of truth? Control of the training dataset could allow for those with an intention to skew results towards their desired outcome to intentionally bias their LLM. Feeding an LLM discredited or fraudulent papers could be an effective tool for spreading misinformation.

### Implications for practice and future research

The integration of ChatGPT into the genetic counseling domain poses both opportunities and challenges. While AI can augment the delivery of genetic education, it cannot yet replace the nuanced understanding and judgment of human professionals. The personalization of responses and the capacity to provide compassionate support are areas where human interaction remains irreplaceable. That is not to say that all interactions will remain this way; research in this area is underway, exploring how generative model responses, under appropriate scenarios, might actually be preferred by patients for their empathetic quality.^58^^,^^59^

Our results show that there is still error in the responses even with the improvements from GPT-4 and that challenges remain in deploying this technology in healthcare. However, this particular chatbot was not necessarily designed to answer questions about medical conditions, and a platform using the same technology that is trained specifically for this task and given rules around genes/disease (autosomal dominant vs recessive inheritance, etc) would likely perform markedly better. As we documented in our earlier study, ChatGPT can provide what appears to be well constructed answers, even when they are wrong (questions 46, 48, and 67). AI generated answers from these search engines may prove even more compelling than the hyperlink-based results from the past, increasing the possibility of people using these results to determine medical care.

Future research should focus on enhancing the accuracy and consistency of AI responses, ensuring the inclusion of the latest scientific data, and exploring ways to balance human-like interaction with the delivery of accurate information. Further studies could also investigate the potential of AI in providing tailored responses to individual queries, taking into account the unique circumstances of each case. Although perfection may never be reached (by humans or AI) in the delivery of rapidly changing genetic information, more work on testing and evaluating the trust and thresholds of accuracy and precision these AI models need to achieve before they should be deployed further into medicine should also be conducted.

### Limitations

Our study had several limitations. First, there are a lack of a validated instruments for evaluating chatbot responses to clinical questions. We identified BUS-15 as an option for this study, but our use of only 7 of the questions reflects this issue. Future potential work in this area could involve the development of such a tool, in addition to continuing to test and monitoring LLM’s ability to assist in medical information dissemination. Second, we tested ChatGPT on relatively common genetic conditions about which there is a large amount of minable information present on the internet. Further studies should probe the ability of this algorithm to adequately perform in rarer or newly discovered conditions, given the rapidly evolving field. Third, this study was conducted with a small team of experts (*n* = 4), results may differ with a larger group of evaluators. However, the small number of LLM studies exploring genetics have also used small groups of evaluators.^15^^,^^16^^,^^60^ Fourth, LLMs by design generate new answers to each query, so there is a chance a slightly different response could be generated on iterative trials. The design of our study prioritized the comparison of responses to similarly structured questions across GPT3.5 and GPT4, rather than iterative submissions of identical prompts. Given this approach, alongside the sophisticated analysis required to definitively assess response variability and the close model of ChatGPT model, we cautiously anticipate that variations in responses between versions may not be markedly different. However, we acknowledge that a comprehensive determination of this expectation would necessitate a more intricate study explicitly designed to evaluate such differences, which is challenged by the proprietary framework of ChatGPT. Additional future work could also include patients to help evaluate the response to genetic queries.

## Conclusion

The emergence and evolution of AI platforms like ChatGPT, particularly in its GPT-4 iteration, underscore a transformative phase in the domain of genomics and healthcare information delivery. While showcasing significant growth and the potential to redefine the landscape of patient education and support, these developments bring forth a dual sentiment of hope and concern. The promise held by AI in revolutionizing the delivery of genomic information, and helping to democratize its access, is as immense as the responsibility it carries.

As AI begins to supplant certain human functions, the need for stringent oversight becomes paramount. This is particularly crucial in the realm of genomics, where misinformation or inaccuracies can lead to critical healthcare decisions being compromised. The incorporation of generalized AI technologies into consumer-facing products exacerbates the need for such oversight. In a domain rife with complexities and varying expert opinions, ensuring the safe and accurate delivery of medical information is a formidable challenge.

To harness the full potential of AI in genomics effectively, it is imperative to establish regulatory guardrails that accommodate diverse viewpoints in medical care while preventing the dissemination of harmful information. The application of continuous updates, expert supervision, and rigorous testing regimes is essential in maintaining the relevance and reliability of AI platforms. Moreover, training these platforms specifically for genetic information dissemination can enable them to augment human interactions in genomics meaningfully.

Looking ahead, platforms like ChatGPT hold tremendous potential to serve as adjunct tools in the field of genetics and genomics. However, this potential can only be realized through a balanced approach that combines technological innovation with ethical responsibility, careful oversight, and a commitment to continuous improvement. As we stand on the brink of this AI-driven transformation, it is imperative to navigate this evolution with a focus on safety, accuracy, and the enhancement of healthcare outcomes. The journey ahead is not just about leveraging AI’s capabilities but also about shaping its role responsibly in a sector as critical as healthcare.

## Supplementary Material

ocae128_Supplementary_Data

## Data Availability

The full dataset with both questions asked to ChatGPT, its responses, and survey responses are available on DataDyrad.org, at https://doi.org/10.5061/dryad.s4mw6m9cv.^44^ The dataset includes prompts and responses from both GPT3.5 and GPT4 studies, and survey responses from the panel of experts.
