# Supplementary material for: A comparative evaluation of ChatGPT 3.5 and ChatGPT 4 in responses to selected genetics questions
Source: J Am Med Inform Assoc. 2024 Jun 14;31(10):2271–83. doi: 10.1093/jamia/ocae128 (PMC11413464; doi:10.1093/jamia/ocae128)
Supplement: ocae128_Supplementary_Data [file ocae128_supplementary_data.zip › ocae128_Supplementary_Data/Supplemental Methods v2.docx]

**Methodological Design for Evaluating LLM Responses in Genetics and Genomics**

**Designing the Evaluation Framework**

Conventional metrics for assessing chatbot performance have predominantly concentrated on factors such as user satisfaction, efficiency, effectiveness, usability, and aesthetic appeal.^1,2^ However, there's a notable gap in validated methodologies for examining clinical outputs provided by these systems.^3^ This gap is particularly pronounced with the advent of generative AI technologies like ChatGPT, which, despite their novelty and complexity, have seen limited representation in scholarly publications due to challenges in their development and deployment. In light of this, we adopted the BOT Usability Scale (BUS-15)^4^ as our primary analytical instrument. The BUS-15, a validated questionnaire encompassing 15 items, was chosen for its emphasis on user satisfaction with AI conversational agents, spanning considerations from functionality and conversation quality to privacy, security, and response timeliness.

From the BUS-15's comprehensive scope, we selected seven items that most directly align with our research objectives, focusing on ChatGPT's handling of medical and genetic inquiries. The reason for not including all of the questions was due to a lack of relevancy that some of the questions posed. For example, we were not evaluating the interface of ChatGPT, so the question about “ease of initiating interactions with the chatbot” was outside of the scope of this study. The selected items were chosen to evaluate the system's capabilities in recognizing and aiding user intentions, relevance, and appropriateness of provided information, informative response without overloading, resilience in handling challenging queries, clarity and politeness of communication, credible and informative response, and inclusivity for a diverse user base, including those with neurodiverse conditions.

To further refine our evaluative approach, we expanded our study to include an analysis of responses generated by the more recent GPT-4 model. This iteration was initiated shortly after concluding our initial work with GPT-3.5, enabling a timely assessment of advancements in generative AI capabilities. Our methodology was adapted to focus specifically on two key areas:

1. **Accuracy of the Answer**: This entailed a thorough evaluation of the factual correctness of responses provided by GPT-4, underscoring the importance of accurate information in clinical contexts.
2. **Relevance of the Information Provided**: We assessed the pertinence of GPT-4's responses to the posed questions, emphasizing the need for AI-generated advice to be directly applicable to the user's inquiry.

In this subsequent phase, we maintained the use of the same BUS-15 questions from our GPT-3.5 study to ensure consistency in our evaluative framework and facilitate a comparative analysis between the two versions of the AI model. However, to streamline the evaluation process and mitigate the time commitment required from our expert reviewers, we omitted three qualitative analysis questions that were part of the initial GPT-3.5 study. This adjustment was based on feedback indicating that the comprehensive review of 68 questions in the GPT-3.5 evaluation demanded approximately 5-6 hours of each expert's time. By refining our approach, we aimed to enhance the efficiency of the evaluation process while preserving the depth and rigor of our analysis.

**Gene selection and Question design**

In the initial phase, a clinical geneticist and two genetic counselors simulated patient interactions with ChatGPT, each focusing on a specific genetic condition (BRCA1, MLH1, or HFE). The selection of BRCA1, MLH1, and HFE as focal points for this study was strategically made due to their significant prevalence and discussion frequency within clinical genetics settings. These genes represent a spectrum of genetic conditions and inheritance patterns, including both autosomal dominant (BRCA1 and MLH1) and autosomal recessive (HFE) traits. This diversity ensures an evaluation of the AI model's ability to handle different genetic counseling scenarios. The professionals crafted over twenty unique queries per gene, designed to mimic the range of questions a patient might pose during genetic counseling. They were instructed to include common straightforward, factually based questions, as well as those requiring subjective judgment or interpretation of complex information that could test the robustness of the ChatGPT response. The authenticity of the inquiry process was preserved by including queries with common errors such as misspellings, without correcting them prior to submission, to evaluate ChatGPT's error-handling capacity. The team of domain experts used to evaluate the responses were selected due to their experience working in a large-scale program to return actionable genetic findings to patients^5^ and an expert outside of the group was also recruited to provide an additional perspective. Author NAW is the clinical geneticist that led the large-scale program and initiated this study as both an author and participant. There were three genetic counselors that were leading different aspects of the large-scale program, all three were contacted via email and invited to participate. One genetic counselor declined participation and the other two accepted the invitation. Two outside expert clinical geneticists were also invited to participate to provide their perspective. One declined and the other accepted the invitation. Prior to performing the evaluation, the team performed a cognitive walkthrough of the study, to help establish guidance on what the Likert scores meant (i.e. what a very poor answer was vs a very strong answer on the quality question).

**Clinical Evaluation of Chatbot Responses**

In the subsequent phase, responses generated by ChatGPT were systematically evaluated using our tailored survey, beginning with one of the selected BUS-15 items followed by the five added questions assessing clinical relevance. Prior to completing the survey, the entire team met on a video conference call to discuss the survey questions. With each survey question, we reviewed the question intention, how to grade it, and in most cases provided examples to help ensure a level of consistency in the response. After addressing the initial set of ChatGPT interactions, evaluators moved on to complete the remaining BUS-15 items. Each response was independently reviewed by a panel comprising two genetic counselors and two clinical geneticists, three of whom had also engaged directly with ChatGPT. This process ensured a thorough and unbiased review of the AI-generated advice, based on both the original queries and the responses.

References

1. Abd-Alrazaq A, Safi Z, Alajlani M, Warren J, Househ M, Denecke K. Technical metrics used to evaluate health care chatbots: scoping review. *Journal of medical Internet research*. 2020;22(6):e18301.

2. Denecke K, Abd-Alrazaq A, Househ M, Warren J. Evaluation Metrics for Health Chatbots: A Delphi Study. *Methods of Information in Medicine*. 2021;60(05/06):171-179.

3. Adamopoulou E, Moussiades L. An overview of chatbot technology. Springer; 2020:373-383.

4. Borsci S, Malizia A, Schmettow M, et al. The Chatbot usability scale: The design and pilot of a usability scale for interaction with AI-based conversational agents. *Personal and ubiquitous computing*. 2022;26(1):95-119.

5. Walton NA, Hafen B, Graceffo S, et al. The development of an infrastructure to facilitate the use of whole genome sequencing for population health. *Journal of Personalized Medicine*. 2022;12(11):1867.
