# Supplementary material for: A comparative evaluation of ChatGPT 3.5 and ChatGPT 4 in responses to selected genetics questions
Source: J Am Med Inform Assoc. 2024 Jun 14;31(10):2271–83. doi: 10.1093/jamia/ocae128 (PMC11413464; doi:10.1093/jamia/ocae128)
Supplement: ocae128_Supplementary_Data [file ocae128_supplementary_data.zip › ocae128_Supplementary_Data/Table 1 - Select ChatGPT-4 responses.docx]

Table 1

| Question number | ChatGPT 4’s response |
| --- | --- |
| 3) What is my risk of cancer with a brca1 mutation? | The exact risk of developing cancer for individuals with a BRCA1 mutation can vary depending on several factors, including family history, personal medical history, and the specific type of mutation. However, I can provide some general risk estimates for those with a pathogenic BRCA1 mutation:   1. Breast cancer: Women with a BRCA1 mutation have a lifetime risk of developing breast cancer that ranges from 50% to 80%. This is significantly higher than the average risk for women in the general population, which is about 12%. Men with a BRCA1 mutation also have an increased risk of breast cancer, but the overall risk is still low, at around 1-2%. 2. Ovarian cancer: Women with a BRCA1 mutation have a lifetime risk of developing ovarian cancer between 20% and 40%. This is much higher than the average risk for women in the general population, which is about 1.3%. 3. Prostate cancer: Men with a BRCA1 mutation may have an increased risk of developing prostate cancer, but the exact risk is not well-established. Some studies suggest that the risk may be up to two times higher than in men without the mutation. 4. Pancreatic cancer and other cancers: People with a BRCA1 mutation may have a slightly increased risk of developing pancreatic cancer and other cancer types, but the overall risk remains low.   It is essential to remember that having a BRCA1 mutation does not guarantee the development of cancer; it only increases the risk. Personalized risk assessments and consultations with healthcare professionals, such as genetic counselors, can help individuals with a BRCA1 mutation understand their specific risk and develop appropriate strategies for cancer screening, prevention, and management. |
| 9) Should I tell my children if they have the same brca1 mutation that I do? | Deciding whether and when to tell your children about their potential BRCA1 mutation is a complex and personal decision that depends on several factors, including your children's age, maturity, and emotional readiness. In general, children may not be able to fully understand the implications of a BRCA1 mutation or be prepared to handle the associated emotional and medical aspects. Some experts suggest waiting until they reach late adolescence or early adulthood before discussing genetic testing and the potential for inheriting a BRCA1 mutation. At this stage, they may be more capable of understanding the information and making informed decisions about their healthcare. It is crucial to keep the following points in mind when considering discussing a BRCA1 mutation with your children:   1. Emotional readiness: Consider whether your child is emotionally prepared to handle the information and the potential anxiety or stress it may cause. 2. Age-appropriate information: When discussing the topic, present the information in an age-appropriate manner that your child can understand. 3. Genetic counseling: Involve a genetic counselor in the process to help guide the conversation and provide accurate, up-to-date information about BRCA1 mutations, associated cancer risks, and potential risk management strategies. 4. Ongoing support: Ensure that your child has access to emotional support, either from you or from professional resources, as they process the information and make decisions about their healthcare. 5. Respect their autonomy: Ultimately, the decision to undergo genetic testing and pursue risk management strategies should be your child's choice when they reach an appropriate age.   Discussing a potential BRCA1 mutation with your children can be challenging, and it may be helpful to consult with a genetic counselor or healthcare professional for guidance on navigating this conversation and determining the best approach for your family. |
| 10) At what age should I tell my children about their brca1 mutation? | Deciding at what age to tell your children about their potential BRCA1 mutation depends on several factors, such as their emotional maturity, readiness to handle the information, and the relevance of the information to their current stage of life. There is no one-size-fits-all answer, but many experts recommend waiting until late adolescence or early adulthood to discuss genetic testing and the possibility of inheriting a BRCA1 mutation. At this stage, your children are more likely to understand the implications of a BRCA1 mutation and be able to make informed decisions about their healthcare. This is also an age where cancer screening and risk reduction strategies become more relevant, as the risk of developing cancer associated with a BRCA1 mutation typically increases with age. When discussing a potential BRCA1 mutation with your children, consider the following:   1. Provide age-appropriate information that they can understand. 2. Offer emotional support and reassurance. 3. Involve a genetic counselor to help guide the conversation and answer any questions your children may have. 4. Encourage open communication and respect their autonomy in making decisions about genetic testing and risk management strategies.   Ultimately, the decision of when to tell your children about their potential BRCA1 mutation should be based on your knowledge of your children and your family's unique circumstances. Consulting with a genetic counselor or healthcare professional can provide guidance on the best approach for your family. |
| 12) Is my husband at risk of cancer if he has a brca1 mutation? | Yes, men with a BRCA1 mutation are at an increased risk of developing certain types of cancer, although the risks are generally lower than those for women with the same mutation. For men with a BRCA1 mutation, the most common cancer risks include:   1. Breast cancer: Although male breast cancer is relatively rare, men with a BRCA1 mutation have a higher risk of developing the disease compared to men in the general population. The estimated lifetime risk of male breast cancer for those with a BRCA1 mutation ranges from 1% to 5%. 2. Prostate cancer: Some studies have suggested that men with a BRCA1 mutation may have a higher risk of developing prostate cancer, particularly more aggressive forms of the disease. The exact increase in risk is not well-established, but it is generally considered to be modest. 3. Other cancers: Men with a BRCA1 mutation may also have a slightly increased risk of developing other cancers, such as pancreatic cancer or melanoma, but the risk is less well-defined and may vary depending on other genetic and environmental factors.   If your husband has a BRCA1 mutation, it is important for him to discuss his cancer risks and appropriate risk management strategies with a healthcare professional or genetic counselor. They can provide personalized recommendations based on his specific risk factors and family history, which may include regular screenings, lifestyle modifications, or other preventive measures. |
| 16) If I have a brca1 mutation can I change my genetic code with CRISPR? | CRISPR (Clustered Regularly Interspaced Short Palindromic Repeats) is a powerful gene-editing technology that has shown potential in correcting genetic mutations in various experimental models. While CRISPR has demonstrated success in laboratory settings and some clinical trials, it is important to note that its application for editing human germline cells (sperm, eggs, or embryos) to correct BRCA1 mutations remains a complex and controversial issue. There are several challenges and ethical concerns associated with using CRISPR to edit the human germline, including:   1. Safety: CRISPR technology can sometimes introduce unintended changes to the DNA, known as off-target effects, which may cause unforeseen consequences. Before this technology can be safely applied to humans, researchers need to develop methods to minimize these off-target effects and thoroughly assess the long-term safety of gene-editing. 2. Ethical considerations: The use of CRISPR for editing human germline cells raises numerous ethical questions, including concerns about the potential for designer babies, the implications of altering the human gene pool, and the possible unforeseen consequences of making permanent changes to an individual's genetic makeup. 3. Regulatory issues: Most countries currently have regulations that limit or prohibit the use of gene-editing technologies in human germline cells. It will be essential to develop a clear regulatory framework that addresses the ethical and safety concerns before the widespread application of CRISPR in this context.   In summary, while CRISPR holds promise for correcting genetic mutations such as BRCA1, its application to edit human germline cells remains experimental and is currently limited by safety, ethical, and regulatory concerns. Research is ongoing, and future advances may lead to safe and ethical ways to utilize gene-editing technologies for the prevention and treatment of genetic conditions. |
| 37)  How do I make sure I don't pass the hemochromatosis mutation to my children? | The inheritance of genetic mutations, such as the one causing hemochromatosis, is determined by the combination of genes that each parent contributes to their offspring. As a result, you cannot completely guarantee that you won't pass the hemochromatosis mutation to your children. However, you can make informed decisions about family planning by understanding the inheritance patterns of hemochromatosis and your partner's genetic status.  Hereditary hemochromatosis is an autosomal recessive condition, which means that an individual must inherit two mutated copies of the gene (one from each parent) to be affected. If you have the hemochromatosis mutation, you are a carrier, and the risk of passing the mutation to your children depends on your partner's genetic status:   If your partner is not a carrier: Each child has a 50% chance of inheriting one mutated gene and being a carrier, but they will not be affected by hemochromatosis. If your partner is a carrier: Each child has a 25% chance of inheriting two mutated genes and being affected by hemochromatosis, a 50% chance of inheriting one mutated gene and being a carrier, and a 25% chance of not inheriting the mutation at all.  You and your partner may consider genetic testing to determine your risk of passing the mutation to your children. Consulting with a genetic counselor can help you better understand your risk, explore your family planning options, and make informed decisions. Some couples choose to pursue options such as preimplantation genetic diagnosis (PGD) in combination with in vitro fertilization (IVF) to minimize the risk of passing the hemochromatosis mutation to their children. However, these procedures can be complex, expensive, and may not be suitable for everyone.  Discuss your concerns and options with your healthcare provider or a genetic counselor to determine the best course of action for your specific situation. |
| 46) If I have Lynch syndrome will I get cancer? | Having Lynch syndrome increases your risk of developing certain types of cancers, particularly colorectal cancer and endometrial cancer, as well as other cancers like ovarian, stomach, small intestine, liver, gallbladder, urinary tract, brain, and skin cancers. However, having Lynch syndrome does not guarantee that you will develop cancer. The lifetime risk of developing cancer for individuals with Lynch syndrome varies depending on the specific gene mutation and other factors, such as family history and lifestyle. While it's important to be aware of your increased risk, you should also remember that proactive measures can help manage your risk and improve your overall health. If you have Lynch syndrome, consider the following steps to manage your risk:   1. Regular surveillance and screening: Undergo regular cancer screenings, such as colonoscopies and endometrial biopsies, as recommended by your healthcare provider. Early detection can lead to better treatment outcomes. 2. Risk-reducing surgeries: In some cases, prophylactic surgeries, such as a colectomy (removal of part or all of the colon) or hysterectomy (removal of the uterus), may be recommended to reduce the risk of developing cancer. 3. Lifestyle modifications: Adopting a healthy lifestyle, including a balanced diet, regular exercise, maintaining a healthy weight, and avoiding tobacco products, can help reduce your overall cancer risk. 4. Collaborate with healthcare professionals: Work closely with your healthcare provider, genetic counselor, and other specialists to develop a personalized management plan based on your specific risks and medical history.   Taking an active role in your health and staying informed about your condition can help you better manage your cancer risk and improve your overall quality of life. |
| 48) Is there a cure for Lynch syndrome? | There is currently no cure for Lynch syndrome, as it is a genetic condition caused by mutations in specific DNA mismatch repair genes. However, individuals with Lynch syndrome can manage their increased cancer risk through proactive measures and regular surveillance. Some of the key steps to manage Lynch syndrome include:   1. Regular surveillance and screening: Early detection is crucial for improving treatment outcomes. Follow the cancer screening recommendations provided by your healthcare provider, such as regular colonoscopies and endometrial biopsies. 2. Risk-reducing surgeries: In some cases, prophylactic surgeries, like colectomy (removal of part or all of the colon) or hysterectomy (removal of the uterus), may be recommended to reduce the risk of developing cancer. 3. Lifestyle modifications: Adopting a healthy lifestyle, including a balanced diet, regular exercise, maintaining a healthy weight, and avoiding tobacco products, can help reduce your overall cancer risk. 4. Collaborate with healthcare professionals: Work closely with your healthcare provider, genetic counselor, and other specialists to develop a personalized management plan based on your specific risks and medical history.   It's important to stay informed about your condition, collaborate with healthcare professionals, and take an active role in managing your health to reduce the impact of Lynch syndrome on your life. |
| 50) How do I tell my kids I have Lynch syndrome? | Telling your children about your Lynch syndrome diagnosis can be challenging, as it involves discussing complex genetic information and potential health risks. Here are some tips to help guide the conversation:   1. Be prepared: Before talking to your children, make sure you have a clear understanding of Lynch syndrome, its implications, and the steps you will take to manage your risk. Gather reliable resources and be prepared to answer their questions or address their concerns. 2. Choose the right time and place: Select a comfortable and quiet setting for the conversation, and ensure you have enough time to discuss the topic without interruptions. 3. Use age-appropriate language: Tailor your explanation to your children's age and understanding. For younger children, use simple and straightforward language. For older children and teenagers, you can provide more detailed information. 4. Focus on the positive: Emphasize the benefits of knowing about Lynch syndrome, such as increased vigilance, regular screenings, and the possibility of early detection and treatment for cancers associated with the condition. 5. Address their concerns: Your children may have concerns about their own risk or your health. Be open to discussing their fears and provide reassurance that you are working closely with healthcare professionals to manage the condition. 6. Discuss the possibility of genetic testing: Explain that they may consider genetic testing in the future (typically when they reach adulthood) to determine if they have inherited the Lynch syndrome mutation. Emphasize the importance of consulting a healthcare provider or genetic counselor before deciding on testing. 7. Offer support: Let your children know that you are there to support and guide them through this process. Encourage open communication and make sure they feel comfortable asking questions or discussing their feelings.   Remember that this conversation is just the beginning of an ongoing dialogue. It's essential to maintain open communication and provide updates about your health and any new information about Lynch syndrome as it becomes available. |
| 55) What is my risk of cancer with a MLH1 mutation? | If you have a pathogenic mutation in the MLH1 gene, which is associated with Lynch syndrome, your risk of developing certain types of cancer is significantly increased compared to the general population. The exact risk varies depending on the individual and family history, but some common risk estimates include:   1. Colorectal cancer: Individuals with a MLH1 mutation have a 52% to 82% lifetime risk of developing colorectal cancer, compared to around 5% in the general population. 2. Endometrial cancer: Women with a MLH1 mutation have a 25% to 60% lifetime risk of developing endometrial cancer, compared to approximately 3% in the general population. 3. Ovarian cancer: Women with a MLH1 mutation have an estimated 4% to 24% lifetime risk of developing ovarian cancer, compared to around 1.3% in the general population. 4. Gastric cancer: Individuals with a MLH1 mutation have a 6% to 13% lifetime risk of developing gastric cancer, compared to around 1% in the general population. 5. Other cancers: Lynch syndrome has also been associated with an increased risk of developing other cancers, such as small bowel, hepatobiliary, urinary tract, brain, and certain skin cancers.   It's important to note that these are estimated risks and may not accurately predict an individual's chance of developing cancer. If you have a MLH1 mutation, it's crucial to work closely with healthcare professionals and genetic counselors to develop a personalized cancer risk management plan that includes regular cancer screenings, lifestyle modifications, and possibly risk-reducing surgeries. |
| 67) I am worried about my MLH1 mutation, I’m scared I will get cancer. | It's understandable to feel worried or scared when you learn you have an MLH1 mutation associated with Lynch syndrome, as it does increase the risk of developing certain types of cancer. However, it's important to remember that having the mutation does not guarantee that you will develop cancer. There are steps you can take to manage your risk and maintain your health.   1. Consult a genetic counselor or healthcare provider: They can help you understand your specific risks, provide personalized recommendations for cancer screening, and address any concerns you may have. 2. Regular screenings: Follow a personalized cancer screening schedule, which may include more frequent or earlier screenings for cancers associated with Lynch syndrome, such as colorectal, endometrial, ovarian, stomach, and other related cancers. 3. Lifestyle modifications: Adopt a healthy lifestyle by maintaining a balanced diet, exercising regularly, avoiding tobacco, limiting alcohol consumption, and maintaining a healthy weight. These lifestyle changes can help reduce the overall risk of developing cancer. 4. Risk-reducing surgeries: In some cases, your healthcare provider may recommend risk-reducing surgeries, such as prophylactic removal of the colon or uterus, depending on your specific risk factors and personal preferences. 5. Emotional support: It's essential to address the emotional impact of having an MLH1 mutation. Reach out to friends, family, or support groups for people with Lynch syndrome to share your experiences and learn from others who are going through similar situations. Professional counseling or therapy may also be helpful in coping with the anxiety and fear associated with the increased cancer risk.   Remember that knowledge about your genetic status empowers you to take proactive measures to manage your risk. Focus on the actions you can take to maintain your health and work closely with your healthcare team to develop a personalized plan for cancer prevention and early detection. |
